# MUC16 stimulates neutrophils to an inflammatory and immunosuppressive phenotype in ovarian cancer

**DOI:** 10.1186/s13048-023-01207-0

**Published:** 2023-08-30

**Authors:** Yuliang Wu, Qi Liu, Yan Xie, Jihui Zhu, Sai Zhang, Yao Ge, Jing Guo, Ning Luo, Wei Huang, Runping Xu, Shupeng Liu, Zhongping Cheng

**Affiliations:** 1 Department of Obstetrics and Gynecology, Shanghai Tenth People’s Hospital, Tongji University, 301 Yanchang Road, Shanghai, 200072 China; 2 Gynecologic Minimally Invasive Surgery Research Center, Tongji University School of Medicine, 1239 Siping Road, Shanghai, 200092 China

**Keywords:** MUC16, Neutrophils, Ovarian cancer, Tumor immune microenvironment, Siglec-9

## Abstract

**Background:**

MUC16 (CA125) is a commonly used tumor marker for ovarian cancer screening and reported to be an immunosuppressive factor by acting on the sialic acid-binding immunoglobulin-like lectin-9 (Siglec-9) on the surface of natural killer cells (NK cells), B cells, and monocytes. However, the role of MUC16 on neutrophils in the tumor microenvironment remains to be further explored.

**Methods:**

The correlation between the proportion and count of peripheral blood cells, serum inflammatory-related factors and serum MUC16 (CA125) level in patients was constructed based on clinical samples. RNAseq data was obtained from TCGA and sequencing of ovarian cancer tissues, followed by TIMER immune cell infiltration and correlation analysis. Ovarian cancer organoid was constructed to stimulate neutrophils with immunophenotype identification by qPCR and flow cytometry. MUC16 protein stimulation to neutrophils validated the role of MUC16 under the analysis of RNA sequencing and inhibition of NK cytotoxicity in vitro.

**Results:**

The serum MUC16 level was positively correlated with the proportion and count of peripheral blood neutrophils, neutrophil-to-lymphocyte ratio (NLR) and inflammatory factors IL-6, IL-8, IL-10 and IL-2R. Siglec-9, the receptor of MUC16, was expressed on neutrophils and was positively correlated to neutrophil infiltration in ovarian cancer. After the stimulation of ovarian cancer organoids and MUC16 respectively, the proportions of CD11b^+^, CD66b^+^, and ICAM-1^+^ neutrophils were significantly increased, while the proportion of CXCR4^+^ neutrophils was slightly decreased, with increasing of of inflammatory factors MMP9, IL-8, OSM, IL-1β, TNF-α, CXCL3, and ROS. RNA-sequencing analysis revealed that inflammatory response, TNFA signaling pathway, and IL6-related pathway were upregulated in MUC16-stimulated neutrophils, accompanied by high expression of immunosuppression-related factors HHLA2, IL-6, TNFRSF9, ADORA2A, CD274 (PD-L1), and IDO1. NK cytotoxicity was decreased when treated by supernanant of MUC16-stimulated neutrophils in vitro.

**Conclusion:**

MUC16 acted on neutrophils by Siglec-9 leading to an inflammatory and immunosuppressive phenotype in ovarian cancer.

**Supplementary Information:**

The online version contains supplementary material available at 10.1186/s13048-023-01207-0.

## Introduction

The incidence of epithelial ovarian cancer (EOC) is increasing, with the 5-year survival rate of patients with advanced ovarian cancer remaining at only 29%, seriously threatening women’s health and life [1, 2]. Nowadays, tumor immunotherapy has been an effective treatment for tumors in addition to surgery, radiotherapy, chemotherapy, and targeted therapy [3, 4]. However, immunotherapy is not effective enough for most ovarian cancer patients due to the suppressive immune microenvironment and the characteristics of “cold” tumors [5, 6]. More exploration of the immune microenvironment of ovarian cancer may help to improve the effectiveness of ovarian cancer immunotherapy.

MUC16 (CA125) is a commonly used tumor marker for EOC screening [7]. Recent research reported that MUC16 could inhibit the anti-tumor activity of the immune cells and cause tumor cell immune escape by acting on the sialic acid-binding immunoglobulin-like lectin-9 (Siglec-9), a brand-new immune checkpoint, on the surface of natural killer cells (NK cells), B cells, and monocytes [8, 9]. Siglec-9 on the T cells can bind to sialylated ligands on the surface of tumor cells, resulting in a significantly increased growth rate of MC38 tumors in mouse [9]. It is suggested that Siglec-9 plays an important role in tumor progression and is one of the potential immunotherapy targets. In vivo experiments have confirmed that sialylated ligands can also act on neutrophils through Siglec-E (the mouse homologous Siglec-9), inhibiting their tumor suppressor activity[10]. Activation of Siglec-9 in non-neoplastic diseases can alter the immunophenotype of neutrophils [11, 12].

It has been reported that chronic inflammation in the tumor microenvironment promotes tumor progression by altering the expression of oncogenes, inhibiting cell apoptosis, promoting angiogenesis, and recruiting suppressive immune cells [13]. IL-6, TGF-β, IL-10 and other pro-inflammatory factors secreted by tumor cells could promote chronic inflammation by stimulating MDSCs, macrophages, and neutrophils to further secret IL-6, TGF-β, IL-10[14]. Chronic inflammation is usually accompanied by the secretion of immunosuppressive factors such as ROS, ARG1, PGE2, PD-L1, IDO1, etc., resulting in promoting the formation of an inhibitory immune microenvironment and inhibiting tumor-killing effect of CD8^+^ T cells and NK cells [13]. As tumor-promoting inflammatory cells, neutrophils is thought to promote the formation of a suppressive tumor immune microenvironment [15]. Relevant molecules in the tumor microenvironment including granulocyte colony-stimulating factor (G-CSF) and transforming growth factor-β (TGF-β) induce an increased secretion of ARG1, ROS, NO, PGE_2_ by neutrophils, thereby inhibiting the activation of CD8 + T cells and NK cells [16]. In addition, a variety of cytokines secreted by neutrophils can recruit activated macrophages, Treg cells, and other immunosuppressive cells, also resulting in tumor immune escape [14, 17–19]. Similar to other immune regulatory cells, immune checkpoint proteins such as PD-L1 and VISTA are expressed on neutrophils and lead to immunosuppression [15]. However, studies on neutrophil infiltration in ovarian cancer and its specific roles and mechanisms in the immune microenvironment are still scarce. There are few studies on the effect of MUC16 on neutrophils to regulate tumor immune microenvironment. The role of MUC16 on neutrophils in the tumor microenvironment remains to be further explored.

Our study found that MUC16 stimulation of neutrophils might be a cause of the systemic hyperinflammatory state in ovarian cancer patients. In vitro experiments and transcriptomic analysis demonstrated that the MUC16 acted on neutrophils by Siglec-9 leading to an inflammatory and immunosuppressive phenotype, with upregulation of inflammatory-related pathways overexpression of immunosuppressive molecules like IL-6 and PD-L1, which inhibited the tumor-killing activity of NK cells.

## Materials and methods

### Biological specimens and data Collection

Ovarian cancer tissues, normal ovarian tissues and blood, and umbilical cord blood were obtained from the Department of Obstetrics and Gynecology, Shanghai Tenth People’s Hospital, Tongji University. The clinic-pathological data of ovarian cancer patients was described in Supplementary Table 1. All the tumor samples were confirmed by experienced pathologists. The study was performed in accordance with the Declaration of Helsinki and was approved by the institutional ethics committee at Shanghai Tenth People’s Hospital. The mRNA expression data of ovarian cancer were obtained from The Cancer Genome Atlas (TCGA) database (https://portal.gdc.cancer.gov/) and our previous study [20].

### Correlation analysis

The immune cell infiltration was calculated on TIMER (http://timer.cistrome.org/) [21, 22]. For Gene Set Variation Analysis (GSVA), we calculated the enrichment score for each sample in the gene set using the R package from GSVA (DOI:10.18129/B9.bioc.GSVA, version 1.40.1) with Molecular Signatures Database (c5.go.bp.v7.4.symbols.gmt) to evaluate the relevant pathways and molecular mechanisms. Correlations were then assessed by the Pearson coefficient and visualized.

### Immunohistochemistry and immunofluorescence

The sections of tumor tissue and organoid were fixed overnight in 4% PFA before paraffin wax professing and embedding. Tissue sections were cut at 4µm size. For immunohistochemical analysis, endogenous peroxidase was blocked with 0.3% hydrogen peroxide for 30 minutes in adjacent sections. Antigen was retrieved using a sodium citrate buffer method by heating at 100°C for 30 minutes. Slides were then incubated with the antibodies for 1 hour. A labeled streptavidin-biotin system with a horse-radish peroxidase label was used to detect the primary antibodies and visualized by incubation with 3,3’-diaminobenzidine chromogen and hydrogen peroxide substrate for 10 min. The slides were then counterstained with hematoxylin and mounted in dibutyl phthalate xylene. For immunofluorescence, tissue/cells were harvested on slides and fixed in 4% PFA. Then, the slides were permeated with 0.5% TritonX-100/PBS for 5 min and blocked with 3% bovine serum albumin. Slides were incubated with antibodies (Supplementary Table 2) overnight at 4 °C. Slides were washed with PBS and incubated with secondary antibody (1:1000 dilutions) for 2 h at RT. Nuclei were counterstained with DAPI (1:1000 dilutions) for 15–30 min.

### Establishment of patient-derived ovarian cancer organoids

Biopsies were obtained from patients with ovarian cancer. The fresh OC tissues were minced and digested with collagenase I (1 mg/ml). The cell suspension was filtered with a 100 μm filter and treated with the erythrocyte lysis solution. Organoids were cultured with the basic medium and the medium was changed every two to three days. Then, cells were centrifuged and resuspended in 75% Matrigel (Corning, USA)/25% complete medium at 2*10^6^/ml. The resuspension was deposited in droplets of 15ul into prewarmed 24-well plates (30ul per well in total) and placed at 37 °C with 5% CO_2_ to solidify for 10 min followed by suspension in the 1ml cell line complete medium (Supplementary Table 2). The medium was changed every 72 h.

### Determination of supernatant components

For MUC16 determination of ovarian cancer organoids supernatant, ELISA was performed following the instructions with the CA125 ELISA kit. Briefly, standard dilution and the supernatant were added into the CA125 ELISA plate and incubate at 37 °C for 1 h, followed by incubation of biotin-conjugated antibody for 1 h at 37 °C, incubation of Streptavidin-HRP for 30 min at 37 °C and incubation of TMB substrate for 15–20 min at 37 °C protected from light. The optical density of each well was measured within 5 min using a microplate reader set to 450 nm and the concentration of MUC16 (CA125) was calculated from the standard curve. For IL-6, IL-8, IL-10, TNF-a determination of neutrophils supernatant, Human Inflammatory Cytokine Cytometric Bead Array (CBA) was used and was performed following the instructions.

### Isolation and activation of primary human neutrophils

Umbilical cord blood (UCB) was collected from cesarean sections in lithium-heparin tubes (Greiner Bio-One 9mL LH, Austria) at Shanghai Tenth People’s Hospital. Informed consent was obtained from the puerperas. Peripheral Blood (PB) collected in lithium-heparin collection tubes were obtained from adult volunteers. Density-gradient centrifugation was used to isolate neutrophils with Histopaque®1077 (Sigma Aldrich, Steinheim, Germany) and Histopaque®1119 (Sigma Aldrich, Steinheim). After erythrocyte lysis, granulocytes were washed once in 1x DPBS (Thermo Fisher, Germany) for 10 min at 800 g and resuspended in RPMI 1640 (Gibco, Germany) supplemented with 10% fetal bovine serum (FBS, Gibco), 100 U/mL Penicillin and 100 µg/mL Streptomycin (Biochrom, Berlin, Germany). The isolated neutrophils were seeded in 24 well (10^6^ cells/well) and cultured at 37℃ with 5% CO2. All described procedures were conducted at room temperature under sterile conditions.

### Neutrophil stimulation

When ovarian cancer organoids are available for co-culture (incubated for approximately 2–3 weeks), umbilical cord blood-derived neutrophils are isolated and were immediately co-cultured with ovarian cancer organoids at a ratio of 1:10 − 1:20 (ovarian cancer cell: neutrophils) for 24 h. Neutrophils were subsequently harvested for subsequent experiments. As for MUC16 stimulation, MUC16 was added to the medium of neutrophils (10^6^ cells/ml) at the concentration of 100ug/ml for 24 h. Neutrophils and the supernatant were subsequently harvested for further experiments and analyses.

### Flow Cytometry and detection of reactive oxygen species (ROS)

For the identification of neutrophil markers, flow cytometry was performed. Neutrophils treated with ovarian cancer organoids or MUC16 for 24 h were washed with ice-cold PBS and blocked with 3% BSA for 20 min, followed by incubation of antibodies for at least 30 min at 4 °C. Then the cells were analyzed on a flow cytometer.

DCFH-DA was used for the detection of reactive oxygen species (ROS). Neutrophils were collected and suspended in DCFH-DA (10 µM) at the concentration of 10^6^/ml and incubated for 20 min in a 37ºC cell incubator. The neutrophils were washed fully to remove the DCFH-DA and detected with flow cytometry under FITC.

### RNA isolation and quantitative real-time PCR

Total RNA was isolated from tissues frozen-sectioned into thin slices and cells using TRIzol reagent (Invitrogen, USA) according to the manufacturer’s protocol. cDNA was synthesized with a FastQuant RT Kit (Tiangen, China). The mRNA expression level was assessed by qRT-PCR with a SuperReal PreMix Plus (SYBR Green) Kit (Tiangen) using a LightCycler 96 (Roche, USA). All mRNA levels were normalized to the levels of GAPDH or β-actin. The primers used in qRT-PCR are listed in Supplementary Table 3.

### Differentially expressed genes (DEGs) and functional enrichment analysis

The data were normalized and analyzed by the DESeq2 package following a previously described method [23]. Genes with a change above 1.5-fold and *P* < 0.05 were considered to be significantly differentially expressed.

We performed functional enrichment analysis utilizing Gene Ontology (GO) enrichment analysis, Kyoto Encyclopedia of Genes and Genomes (KEGG) pathway enrichment analysis, and Hallmarks enrichment analysis with the subset of background files downloaded from the Molecular Signatures Database (http://www.gsea-msigdb.org/gsea/downloads.jsp) as c2.cp.kegg.v7.4.symbols.gmt, c5. go.bp.v7.4.symbols.gmt, and h.all.v7.4.symbols.gmt, respectively. The results were visualized with Circos. For Gene set enrichment analysis (GSEA), we divided the samples into two groups according to whether they were treated by MUC16 to evaluate the relevant pathways and molecular mechanisms based on GO BP functional annotation, KEGG pathway enrichment analysis, and Hallmarks enrichment analysis. The minimum gene set was set to 5 and the maximum gene set to 5000 based on gene expression profiles and phenotypic groupings, and one thousand resamplings with P value < 0.05 and FDR < 0.25 were considered statistically significant.

### NK cell killing assay

LDH kit was performed to detect NK cell killing efficiency, and according to the instructions. OVCAR3 was seeded into 96-well cell plates at a density of 5000 cells per well and NK-92 cells were treated with IL-2 starvation for 4 h in advance, followed by pretreatment with neutrophil supernatant, MUC16-treated neutrophil supernatant and MUC16-contained medium for 4 h. Fresh culture medium (without IL2) was changed and NK cells were co-cultured with OVCAR3 (E:T = 1:1). LDH was detected by a microplate reader after 6 h and the killing efficiency of NK-92 was calculated according to the instructions.

### Statistics

The data are presented as the mean ± SEM unless otherwise noted. The significance of differences was evaluated with Student’s t-test. All analyses were performed with GraphPad Prism. When the *P*-value was < 0.05, the results were considered to be statistically significant.

## Results

### MUC16 and its receptor were associated with inflammation and neutrophil infiltration in ovarian cancer

**Fig. 1:**
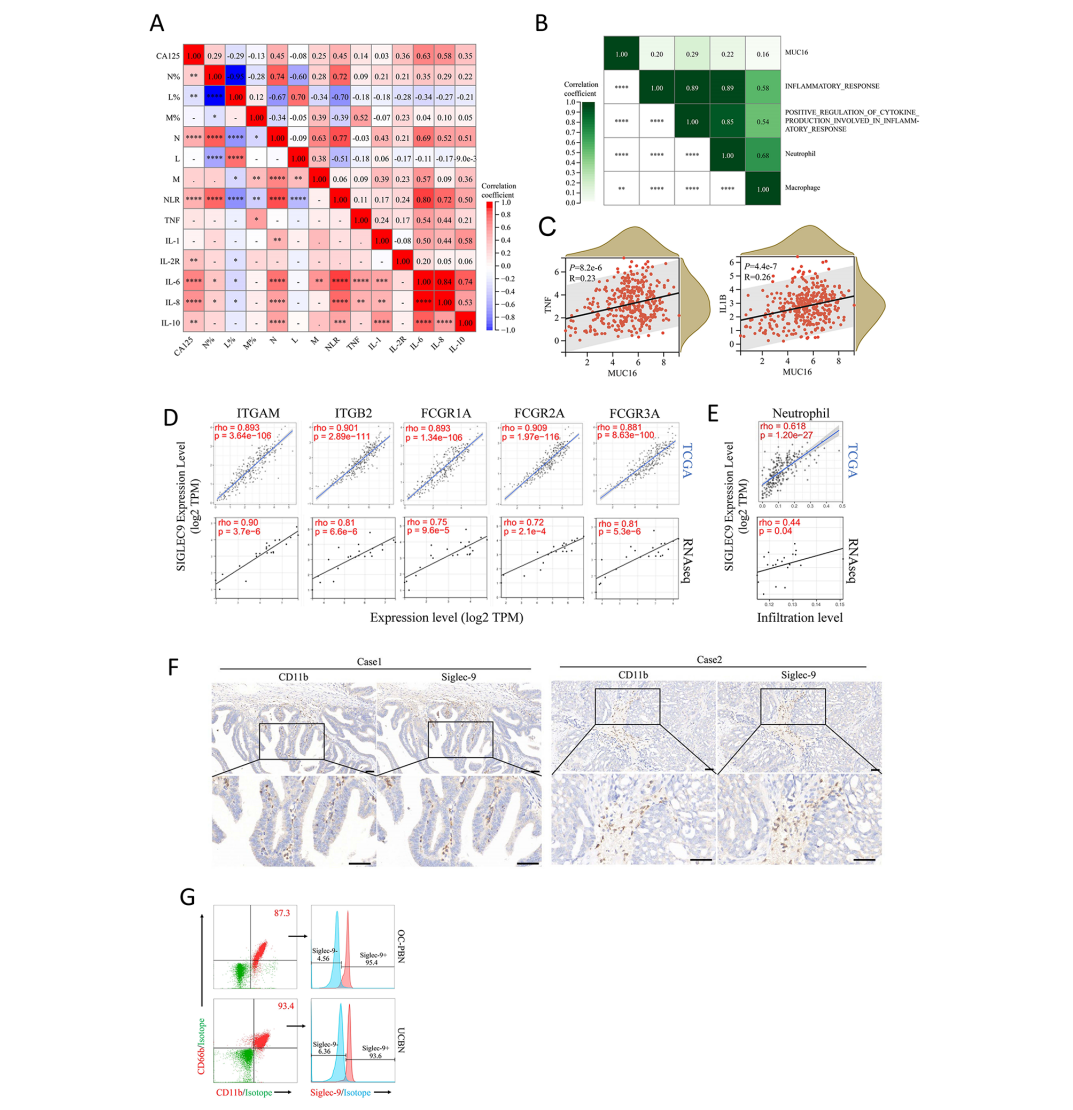
MUC16 and Siglec-9 were associated with inflammation and neutrophil infiltration in ovarian cancer. **(A)** The correlation between the proportion and count of peripheral blood cells, serum inflammatory-related factors and serum MUC16 (CA125) level in ovarian cancer patients (n = 99). (N: count of peripheral blood neutrophil; N%: proportion of peripheral blood neutrophil; L: count of peripheral blood lymphocytes; L%: proportion of peripheral blood lymphocytes; M: count of peripheral blood monocytes; M%: proportion of peripheral blood monocytes) **(B)** The correlation among MUC16, inflammation pathways, neutrophils, and macrophages in ovarian cancer according to TCGA Ovarian Cancer (TCGA-OV) dataset with GSVA. **(C)** The correlation between MUC16 and the inflammatory factors IL-6 and IL-1B expression according to TCGA-OV. **(D-E)** The correlation between Siglec-9 and the markers of neutrophils **(D)** and neutrophil infiltration **(E)** based on TCGA-OV database and ovarian cancer tissue RNA sequencing data. **(F)** Immunohistochemical staining on serial sections of ovarian cancer tissue with CD11b and Siglec-9 in two different patients (Case 1 and Case 2). **(G)** Detection of the expression of Siglec-9 of neutrophils from peripheral blood of ovarian cancer patients and umbilical cord blood by flow cytometry. (A-C: R = Pearson’s correlation; D-E: rho = Spearman correlation; NLR: neutrophil-to-lymphocyte ratio. PBN: peripheral blood neutrophils; UCBN: umbilical cord blood-derived neutrophils; *p < 0.05, **p < 0.01, ***p < 0.001, ****p < 0.0001.)

Inflammation is an important role in the initiation and development of epithelial ovarian cancer (EOC). We first explored the correlation between the proportion and count of peripheral blood cells, serum inflammatory-related factors and serum MUC16 (CA125) level in patients (Fig. 1A). The results showed that the serum MUC16 level was negatively correlated with the proportion and count of peripheral blood lymphocytes, and positively correlated with the proportion and count of peripheral blood neutrophils and neutrophil-to-lymphocyte ratio (NLR) (Fig. 1A). In addition, serum MUC16 level was also positively correlated with inflammatory factors IL-6, IL-8, IL-10 and IL-2R (Fig. 1A). These results suggested that MUC16 might be positively correlated with neutrophil-induced inflammation.

We then explored the correlation between MUC16 and inflammation and key inflammatory cells in ovarian cancer according to TCGA Ovarian Cancer dataset with GSVA. The results showed that MUC16 expression was positively correlated to inflammatory response and positive regulation of cytokine production involved in inflammatory response (Fig. 1B). Correlation analysis also suggested a positive correlation of MUC16 with the inflammatory factors TNF and IL-1B (Fig. 1C).

Since a stronger correlation was observed between MUC16 and neutrophils compared to macrophages, with a stronger correlation to inflammatory pathways (Fig. 1B), we speculated that MUC16 might be involved in neutrophil-induced inflammation in ovarian cancer. Siglec-9, the receptor of MUC16, had strong correlations with markers of neutrophil ITGAM, ITGB2, FCGR1A, FCGR2A, FCGR3A, analyzed with TCGA-OV database and ovarian cancer tissue RNA sequencing data (Fig. 1D). The infiltration levels of neutrophils were also positively correlated to Siglec-9 expression (Fig. 1E). We then verified the infiltration of neutrophils in ovarian cancer tissues with CD11b, and CD66b (Supplementary Fig. 1A-B). Immunohistochemical staining on serial sections of ovarian cancer tissue with CD11b and Siglec-9 showed that the positions of CD11b^+^ cells and Siglec-9^+^ cells in adjacent sections were highly overlapped (Fig. 1F). We collected neutrophils from the peripheral blood of ovarian cancer patients and umbilical cord blood and detected the expression of Siglec-9 by flow cytometry. The results showed that both peripheral blood and cord blood-derived neutrophils expressed Siglec-9, with expression percentages of 95.4% and 93.6%, respectively (Fig. 1G). Immunofluorescence results also showed that most blood-derived neutrophils expressed Siglec-9 (Supplementary Fig. 1C).

### The phenotype of neutrophils was altered by stimulation of ovarian cancer organoids

**Fig. 2:**
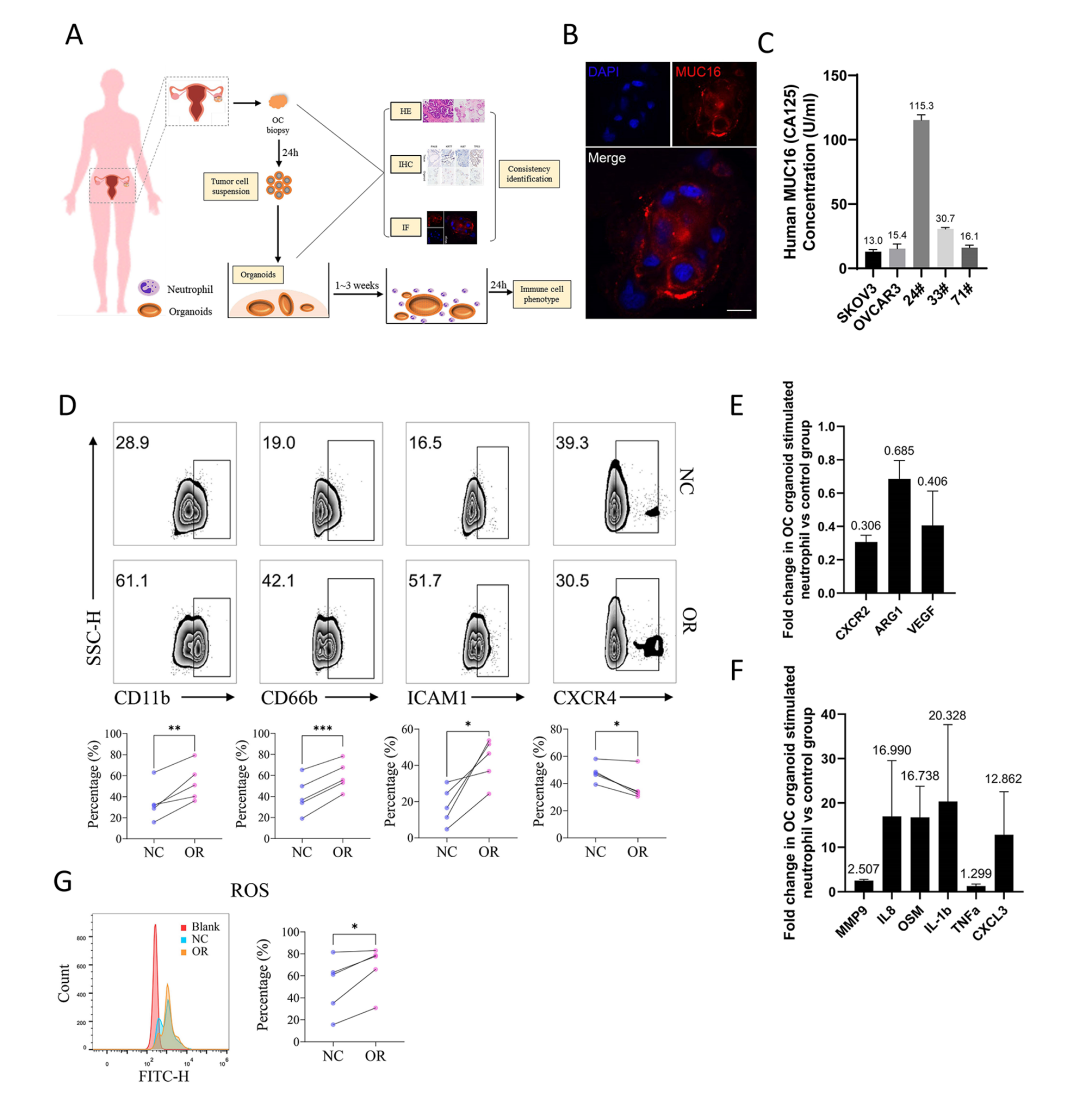
Patient-derived ovarian cancer organoids altered the immune phenotype of neutrophils. **(A)** Flow chart for the establishment of patient-derived ovarian cancer organoids. **(B)** Expression of MUC16 in ovarian cancer organoids (24#) determined by immunofluorescence under the confocal microscope, bar = 10 μm. **(C)** MUC16 level in the supernatants of ovarian cancer cell lines and ovarian cancer organoids detected by ELISA assay. **(D)** The proportion of CD11b^+^, CD66b^+^, ICAM-1^+^, CXCR4^+^ neutrophils in the ovarian cancer organoid stimulation group (OR) and control group (NC). The upper panel shows the representative flow cytometry results; the lower panel shows the experimental results of 5 cases of neutrophils from different patients. Paired t-test. **(E-F)** The expression of function-related factors in the ovarian cancer organoid stimulation group compared to the control group determined by qPCR (three independent replicate experiments of neutrophils derived from different patients). Error Bar = Mean ± SEM. **(G)** ROS detection of ovarian cancer organoid stimulation group (OR) and control group (NC). Representative results were shown on the left. Blank: blank control group without DCFH-DA; NC: negative control group without ovarian cancer organoid stimulation; OR: ovarian cancer organoid stimulation group. N = 5, paired t-test. (*p < 0.05, **p < 0.01, ***p < 0.001, ****p < 0.0001.)

To explore the effect of ovarian cancer on neutrophils, we established a co-culture system of neutrophils and ovarian cancer organoids (OCOs), an in vitro model that could well mimic in vivo tumors (Fig. 2A and Supplementary Fig. 2A). We used HE staining and immunohistochemical staining of molecular markers such as TP53 and PAX8 to verify the consistency of OCOs with the source tissue (Supplementary Fig. 2B). The OCOs faithfully inherited the molecular characteristics of the derived tumor tissues, with high MUC16 (CA125) expression verified by immunofluorescence under confocal microscopy (Fig. 2B) and ELISA (Fig. 2C). Then, the neutrophils were co-cultured with 24# OCOs for 24-h stimulation. The neutrophils were observed to converge to the vicinity of OCOs (Supplementary Fig. 2C).

To investigate the altered immunophenotype of neutrophils, flow cytometry was subsequently applied to detect the expression of the degranulation markers CD11b (ITGAM) and CD66b (CEACAM8) on the surface of neutrophils, as well as ICAM-1 (CD54) and CXCR4 (CD184), which were commonly used as indicators of tumor-associated neutrophils phenotypes. The results showed that the proportions of CD11b^+^, CD66b^+^, and ICAM-1^+^ neutrophils were significantly increased, while the proportion of CXCR4^+^ neutrophils was slightly decreased after the stimulation of OCOs (Fig. 2D). To further explore the changes in the immunophenotype of neutrophils, we used qPCR to detect genes related to neutrophil function. The results showed that, after stimulation by OCOs, the expression of CXCR2 (0.306 ± 0.041), ARG1 (0.685 ± 0.111), and VEGF (0.406 ± 0.206) were decreased (Fig. 2E), while inflammatory factors MMP9 (2.507 ± 0.287), IL-8 (16.990 ± 12.561), OSM (16.738 ± 7.043), IL-1β (20.328 ± 17.312), TNF-α (1.299 ± 0.442), and CXCL3 (12.862 ± 9.681) were increased in different degrees in neutrophils (Fig. 2F). Since ROS is an important inflammatory factor for neutrophils to regulate the immune microenvironment and affect tumor progression in the tumor microenvironment, we assessed the production of ROS in neutrophils. The results showed that ROS production was significantly increased in neutrophils after stimulation of OCOs (Fig. 2G). In addition, we found a significant increase in the proportion of Siglec-9^+^ neutrophils after stimulation by ovarian cancer organoids, suggesting that Siglec-9 might play a role in the effect of ovarian cancer cells on neutrophils (Supplementary Fig. 2D).

### MUC16-stimulated neutrophils showed a similar phenotype to ovarian cancer organoid-stimulated neutrophils

**Fig. 3:**
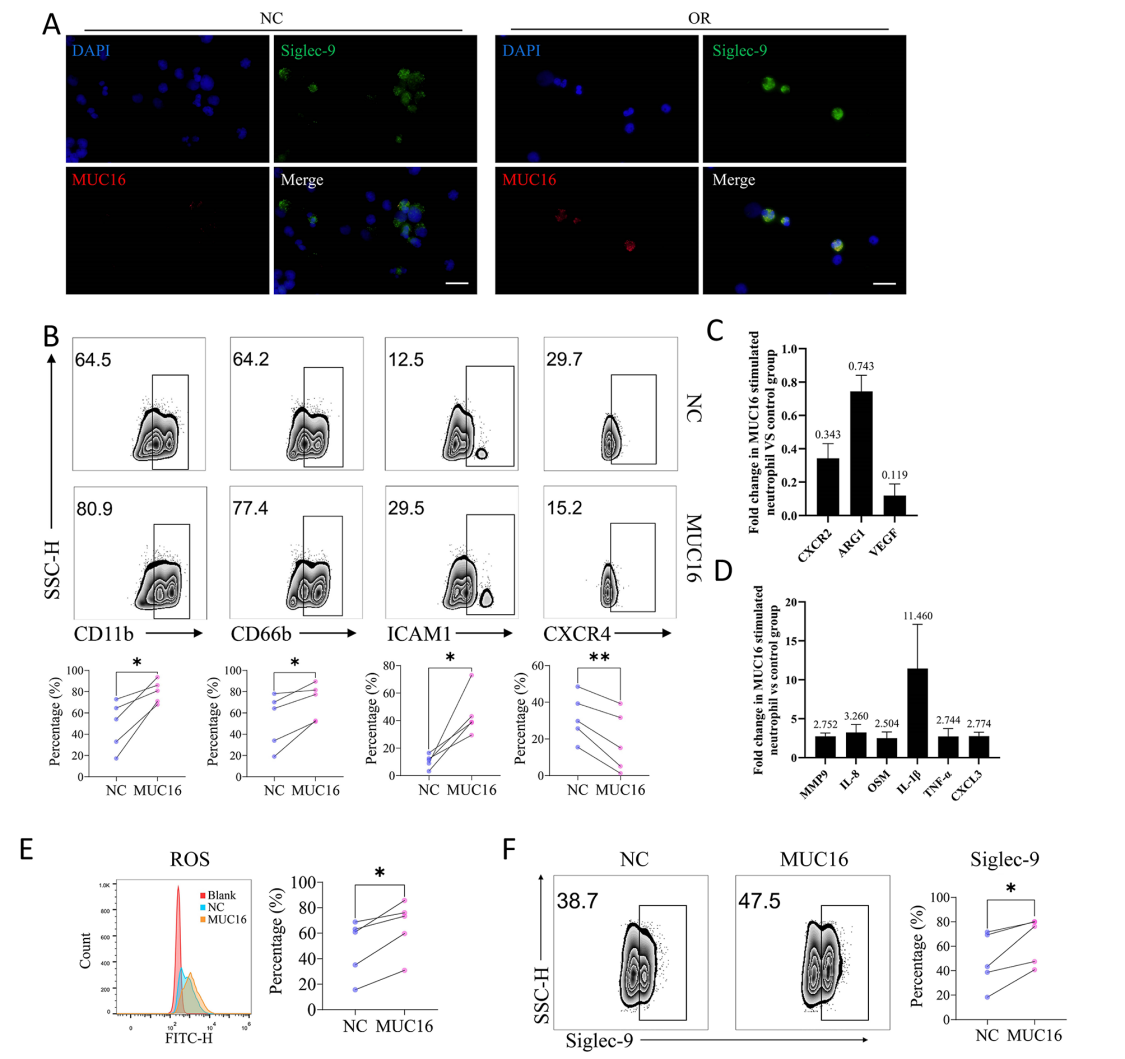
MUC16 altered the immune phenotype of neutrophils similar to ovarian cancer organoids. **(A)** Immunofluorescence staining showed MUC16 adhesion to Siglec-9^+^ neutrophils after stimulation by ovarian cancer organoids. NC: negative control group without ovarian cancer organoid stimulation; OR: ovarian cancer organoid stimulation group. Bar = 50 μm. **(B)** The proportion of CD11b^+^, CD66b^+^, ICAM-1^+^,CXCR4^+^ neutrophils in the MUC16 stimulation group (MUC16) and control group (NC). The upper panel shows the representative flow cytometry results; the lower panel shows the experimental results of 5 cases of neutrophils from different patients. Paired t-test. **(C-D)** The expression of function-related factors in the MUC16 stimulation group compared to the control group determined by qPCR (three independent replicate experiments of neutrophils derived from different patients). Error Bar = Mean ± SEM. **(E)** Detection of ROS by flow cytometry with DCFH-DA stimulation. Representative results are shown on the left. Blank: blank control group without DCFH-DA; NC: negative control group; MUC16: MUC16 treatment group. N = 5, paired t-test, *p < 0.05. **(F)** The flow cytometry results showed the proportion of Siglec-9^+^ neutrophils in the MUC16-treated group (MUC16) and the control group (NC). The left panel shows the representative flow cytometry results; the right panel shows the experimental results of 5 cases of neutrophils from different patients. Paired T-test, *p < 0.05

It has been previously reported that Siglec-9 expressed on the surface of immune cells is a receptor for MUC16 and that the binding of MUC16 to Siglec-9 can alter the phenotype of immune cells [8, 12, 24]. Considering that MUC16 and its receptor were associated with inflammation and neutrophil infiltration in ovarian cancer, we speculated that the stimulation of MUC16 might be responsible for the alterations of neutrophil immunophenotype. To verify whether MUC16 could bind to neutrophils after co-cultured with OCOs, immunofluorescence staining was performed and the results showed that Siglec-9^+^ neutrophils had MUC16 protein adhesion while Siglec-9^−^ neutrophils did not (Fig. 3A), suggesting that MUC16 protein could bind Siglec-9.

To explore whether the alterations in the immunophenotype of neutrophils induced by ovarian cancer organoids were mediated by MUC16, we treated neutrophils with 100 µg/ml MUC16 protein and examined the immunophenotype of neutrophils. The results showed that similar to the stimulation of ovarian cancer organoids, the proportions of CD11b^+^, CD66b^+^, and ICAM-1^+^ neutrophils in each case of neutrophils were significantly increased after MUC16 treatment for 24 h, while the proportions of CXCR4^+^ neutrophils were decreased slightly (Fig. 3B). The qPCR results showed that, after MUC16 stimulation, the expression of CXCR2 (0.306 ± 0.041), ARG1 (0.685 ± 0.111), and VEGF (0.406 ± 0.206) (Fig. 3C), while inflammatory factors MMP9 (2.507 ± 0.287), IL-8 (16.990 ± 12.561), OSM (16.738 ± 7.043), and IL-1β (20.328 ± 17.312), TNF-α (1.299 ± 0.442), and CXCL3 (12.862 ± 9.681) were increased (Fig. 3D), and the results were similar to that of OCOs stimulation (Fig. 2E-F). ROS production was also significantly increased in neutrophils after MUC16 stimulation (Fig. 3E). Similarly, the proportion of Siglec-9^+^ neutrophils was significantly increased after MUC16 stimulation (Fig. 3F).

### MUC16-stimulated neutrophil showed an inflammatory and immunosuppressive phenotype

**Fig. 4:**
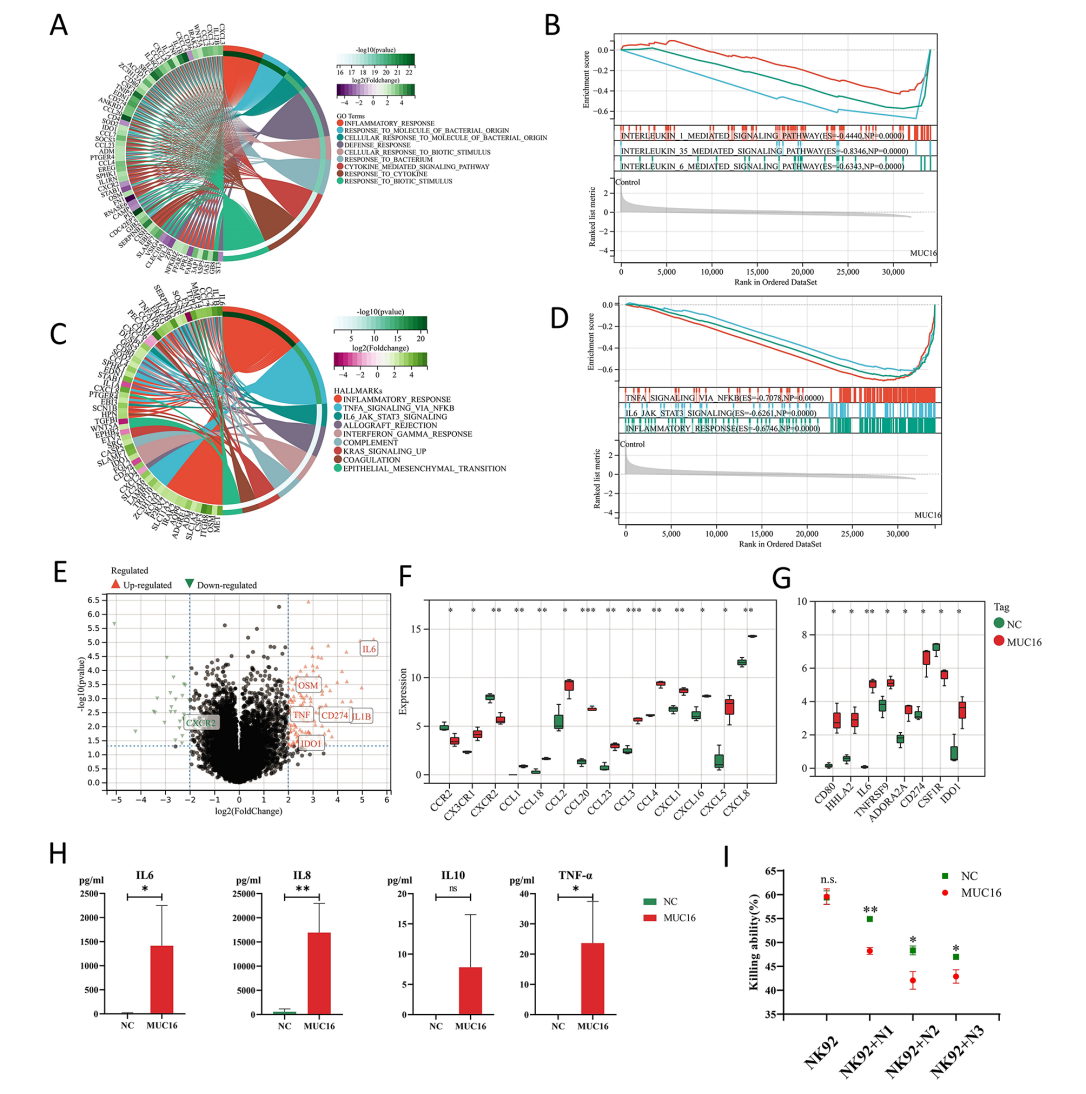
MUC16-stimulated neutrophils showed an inflammatory and immunosuppressive phenotype. **(A)** Circos of GO BP enrichment analysis of differentially expressed genes in the MUC16 treatment group compared with the control group. **(B)** Results of GSEA analysis according to GO BP in the MUC16-treated group compared to the control group. **(C)** Circos of Hallmarks enrichment analysis of differentially expressed genes in the MUC16 treatment group compared to the control group. **(D)** Results of GSEA analysis according to Hallmarks in the MUC16 treated group compared to the control group. **(E)** Volcano plot of differentially expressed genes (|log2FC|>2, p < 0.05) in the MUC16-treated group compared to the control group. **(F)** Expression of chemokines and receptors in the MUC16-treated group (MUC16) and the control group (NC). Student’s t-test, *p < 0.05, **p < 0.01, ***p < 0.001. **(G)** The expression of immunoregulatory factors in the MUC16 treatment group (MUC16) and the control group (NC). Student’s t-test, *p < 0.05, **p < 0.01. **(H)** The levels of IL-6, IL-8, IL-10, and TNF-α secreted by neutrophils in the MUC16-treated group (MUC16) and the control group (NC). Student’s t-test, ns: none significance, *p < 0.05, **p < 0.01. Error Bar = Mean ± SEM. **(I)** Effect of neutrophil supernatant treatment on NK-92 killing of OVCAR3. N1/2/3 are the culture supernatants of neutrophils from different patients, respectively. Student’s t-test, n.s. none significance, *p < 0.05, **p < 0.01. Error Bar = Mean ± SEM.

To detect the changes in the expression profile of neutrophils stimulated by MUC16, we identified differentially expressed genes (DEGs) between the MUC16-treated group and the control group by transcriptome sequencing. Compared with the control group, a total of 172 genes were differentially expressed in the MUC16-treated group, of which 143 genes were up-regulated and 29 genes were down-regulated (Supplementary Fig. 3A). Then we performed GO (Gene Ontology) biological process (BP) functional annotation, KEGG pathway enrichment analysis and Hallmarks enrichment analysis on the differential genes. The results of GO BP functional annotation analysis showed that differentially expressed genes were mainly enriched in inflammatory responses, immune responses to bacteria, immune responses to cytokines, etc. (Fig. 4A). Hallmarks enrichment analysis suggested that the differential genes were mainly enriched in inflammatory response, NFKB-mediated TNFA signaling, and IL6-JAK-STAT3 pathway (Fig. 4B). The results of KEGG pathway enrichment analysis showed that the differential genes were mainly enriched in cytokine-cytokine receptor interaction, IL-17 signaling pathway, TNF signaling pathway and chemokine signaling pathway (Supplementary Fig. 3B).

We also performed a GSEA analysis and the results according to GO BP showed that genes related to IL-1, IL-35 and IL-6-mediated signaling pathways were highly expressed in the MUC16-treated group (Fig. 4C). GSEA enrichment results based on Hallmarks showed that NFKB-mediated TNFA signaling, IL6-JAK-STAT3 pathway and inflammatory response pathway were up-regulated in the MUC16-treated group (Fig. 4D). GSEA enrichment results based on KEGG showed that the MUC16 treatment group up-regulated JAK-STAT signaling pathway, TOLL-like receptor signaling pathway and chemokine signaling pathway (Supplementary Fig. 3C). The results above showed that MUC16 treatment led to the upregulation of inflammatory-related pathways such as interleukin-related pathways, TNF signaling pathways, and Toll-like receptor signaling pathways in neutrophils, and induced neutrophil inflammatory responses.

Chronic inflammation plays an important role in the formation of a tumor-suppressive immune microenvironment, making immune cells exhibit an immunosuppressive phenotype, including expression alterations in chemokines and receptors, immune costimulatory molecules, immune checkpoints, cell factors, etc [13]. In our results, MUC16-treated neutrophils significantly up-regulated OSM, TNF, IL1B, IL-6 and other pro-inflammatory factors, and CD274 (PD-L1), IDO1 and other immunosuppressive-related factors (Fig. 4E). According to our results, CCL1, CCL18, CCL2, CCL20, CCL23, CCL3, CCL4, CXCL1, CXCL16, CXCL5, CXCL8 (IL-8), and CX3CR1 were significantly upregulated, and CCR2, CXCR2 was downregulated (Fig. 4F, Supplementary Fig. 3D).

In addition, the immune regulators CD80, HHLA2, IL-6, TNFRSF9, ADORA2A, CD274 (PD-L1), and IDO1 were significantly up-regulated in the MUC16-treated group, while CSF1R was highly expressed in the control group (Fig. 4G). The upregulation of various immunosuppressive factors suggested that MUC16 treatment of neutrophils resulted in their promotion of the formation of an inhibitory immune microenvironment. We also collected the culture supernatant of neutrophils and detected the levels of IL-6, IL-8, IL-10 and TNF-α secreted by neutrophils. The results showed that the levels of IL-6, IL-8, and TNF-α secreted by neutrophils treated with MUC16 were significantly increased, and the secretion of IL-10 was slightly increased (Fig. 4H). Since IL-6, IL-8, PD-L1, IDO and ROS have been shown to inhibit the killing function of NK cells [25], we treated NK-92 cells with the supernatant of MUC16- treated neutrophils and evaluated the ability of NK-92 to kill ovarian cancer cell line OVCAR-3. The results showed that the killing ability of NK-92 was weakened after treatment with the neutrophil supernatant, but the killing ability of NK-92 was more significantly inhibited after treatment with the supernatant of MUC16-treated neutrophils (Fig. 4I).

## Discussion

MUC16 (CA125) is commonly used as a tumor marker for EOC screening. Our study found positive correlations between the serum MUC16 levels and the number of neutrophils and the inflammatory factors in ovarian cancer patients (Fig. 1), indicating that MUC16 was correlated to the inflammatory and immune microenvironment. Recent studies have revealed that MUC16 played an important role in Siglec-9-mediated tumor cell immune escape [8, 9]. Our study revealed that Siglec-9 was correlated to neutrophil related-markers expression and its infiltration by TCGA dataset and tumor RNAseq data analysis (Fig. 1E-F) and verified that Siglec-9 existed on neutrophils (Fig. 1G-H). These results suggested a possible correlation between MUC16 and neutrophil-associated inflammation in ovarian cancer patients.

The role of tumor-associated neutrophils (TANs) in the tumor immune microenvironment has received increasing attention in recent years [15, 26, 27]. Neutrophils have distinct functions and are defined as tumor-inhibiting (N1) neutrophils, tumor-promoting (N2) neutrophils, and polymorphonuclear neutrophil myeloid-derived suppressor cells (PMN-MDSCs). Identification of different types of neutrophils largely depends on their functional phenotype, with few specific cell surface markers [15]. CD11b (ITGAM) and CD66b (CEACAM8), as surface molecular markers of neutrophil degranulation, were highly expressed after ovarian cancer organoids stimulation, which indicated that ovarian cancer cells could activate neutrophils (Fig. 2D). ICAM1^+^ neutrophils are generally considered to have anti-cancer effects while CXCR4^+^ neutrophils exert the opposite effect [28]. In this study, the proportion of CXCR4^+^ neutrophils decreased after stimulation by OCOs, but the proportion of ICAM1^+^ neutrophils increased (Fig. 2D). The results indicated that neutrophils might present a tumor suppressor phenotype. However, we also observed a decrease in other tumor-suppressive factors and an increase in tumor-promoting markers. The expression of ARG1 and VEGF, which could inhibit CTL cell function [29–31], was reduced in neutrophils stimulated by OCOs (Fig. 2E). These results demonstrated the complexity of the role of neutrophils in ovarian cancer. In addition, neutrophils can also secrete bioactive substances that promote tumor progression, including VEGF, MMP9, Oncostatin M (OSM), IL-1β, ​​TNF-a, IL-8, CXCL3, ROS [31–36]. Our results showed that pro-inflammatory and tumor-promoting factors MMP9, IL-8, OSM, IL-1β, TNF-α, CXCL3, and ROS all increased to varying degrees after OCOs stimulation (Fig. 2F-G). ROS was previously thought to be involved in direct tumor killing by neutrophils [37], however, recent studies have shown that ROS generated by neutrophils can inhibit T cells and NK cells killing tumor cells [38–40]. The results showed that OCOs induced immunophenotype alteration of neutrophils. However, due to the complexity of the tumor microenvironment, especially the immune regulatory network, the role of neutrophils might be multifaceted, which means they might be in an intermediate state of tumor-promoting and tumor-suppressing. Further study of neutrophils might be able to modulate them to be a part of tumor suppression.

Mucins abnormally highly expressed in various tumors are generally considered to be related to tumor metastasis [41]. Recent studies have shown that mucins could regulate the tumor immune microenvironment inducing immunosuppression by interacting with the Siglec family on the surface of immune cells [41]. Siglec-9 has been confirmed as a ligand of MUC16 by multiple studies [8, 11, 12, 24, 42] and has been shown to be expressed on the surface of a variety of immune cells including neutrophils. We also observed MUC16 adhesion on Siglec-9^+^ neutrophils (Fig. 3A). In addition, Siglec-9 was significantly positively correlated with neutrophil-related markers and neutrophil infiltration in ovarian cancer according to the analysis of TCGA dataset and tissue sequencing data (Fig. 1D-E). Considering the results of OCOs stimulation, we assumed that MUC16 expressed by ovarian cancer altered neutrophils’ immunophenotype. Subsequent stimulation of neutrophils by MUC16 resulted in a similar neutrophils immunophenotype to the OCOs stimulation (Fig. 3B-F), suggesting that MUC16 might be involved in the activation and maintenance of neutrophil function. In addition, both OCOs and MUC16 stimulation resulted in the increasement of Siglec-9^+^ neutrophils and high expression in neutrophils (Supplementary Fig. 2D, Fig. 3F, Supplementary Fig. 4E), indicating that there might be a positive feedback effect on the MUC16-Siglec-9 pathway. Although the above results could not indicate that MUC16 stimulation of neutrophils led to a clear phenotype, we observed a significantly increased expression of pro-inflammatory factors in MUC16-stimulated neutrophils, such as MMP9, IL-8, OSM, IL-1β, TNF-α, CXCL3 (Fig. 3D). Analysis of the serum samples and TCGA datasets also suggested positive correlations among MUC16, neutrophils, and inflammatory factors or inflammatory pathways (Fig. 1A-C). Chronic inflammation caused by long-term infiltration of neutrophils is one of the reasons for the formation of a suppressive immune microenvironment [36, 43]. Our study showed that MUC16 stimulation activated inflammatory-related pathways such as interleukin-related pathways, TNF signaling pathways, and Toll-like receptor signaling pathways in neutrophils (Fig. 4A-D), thereby causing downstream functional changes and secretion/expression of IL-6, IL-8, IL-10, PD-L1, IDO, ROS, etc. (Fig. 4E-G). IL-6 could in turn promote the tumor-promoting function, prolong the lifespan, and maintains the activated state of neutrophils [44]. In the tumor microenvironment, IL-6 can also promote the immunosuppressive functions of MDSCs and macrophages, and can directly inhibit NK cells and CD8^+^ CTL cells, resulting in a tumor-suppressive immune microenvironment [25, 45, 46]. The high expression of IDO1 and immune checkpoints PD-L1 and PD-L2 can inhibit the killing effect of CD8^+^ CTL cells and NK cells. In our study, MUC16 induced high expression of immunosuppressive factors such as IL-6, IL-8, PD-L1 and IDO1 in neutrophils, which might result in the weakness of tumor cell-killing ability for NK cells (Fig. 4H-I).

Although we validated that MUC16 induced an inflammatory response in neutrophils, our study still left much to be desired. The neutrophil-centered regulatory network still needs further experimental confirmation and the complex immune regulatory mechanisms cannot be verified by in vitro experiments alone and need to be further investigated by more rigorous in vivo experiments. Furthermore, it is well known that the immune microenvironment of tumors is quite complex and consists of numerous immune cells. The stronger affinity of MUC16 with specific immune cells expressing Siglec-9 may change the specific immune microenvironment and provide new directions for tumor immunotherapy, which remains to be further studied.

In conclusion, our study found that MUC16 induced an inflammatory response in neutrophils, which promotes the development of a systemic hyperinflammatory state in ovarian cancer patients. Factors upregulated by neutrophils’ inflammatory response would lead to an immunosuppression tumor microenvironment and inhibit NK cells. The understanding of the immunoregulatory role of neutrophils would provide ideas and a basis for the treatment of ovarian cancer with neutrophils as target cells in the future.

### Electronic supplementary material

Below is the link to the electronic supplementary material.

Supplementary Material 1

Supplementary Material 2
