# Supplementary material for: MUC16 stimulates neutrophils to an inflammatory and immunosuppressive phenotype in ovarian cancer
Source: J Ovarian Res. 2023 Aug 30;16:181. doi: 10.1186/s13048-023-01207-0 (PMC10466733; doi:10.1186/s13048-023-01207-0)
Supplement: Supplementary file 2 — Supplementary Material 2 [file 13048_2023_1207_MOESM2_ESM.docx]

**Supplementary Figures**


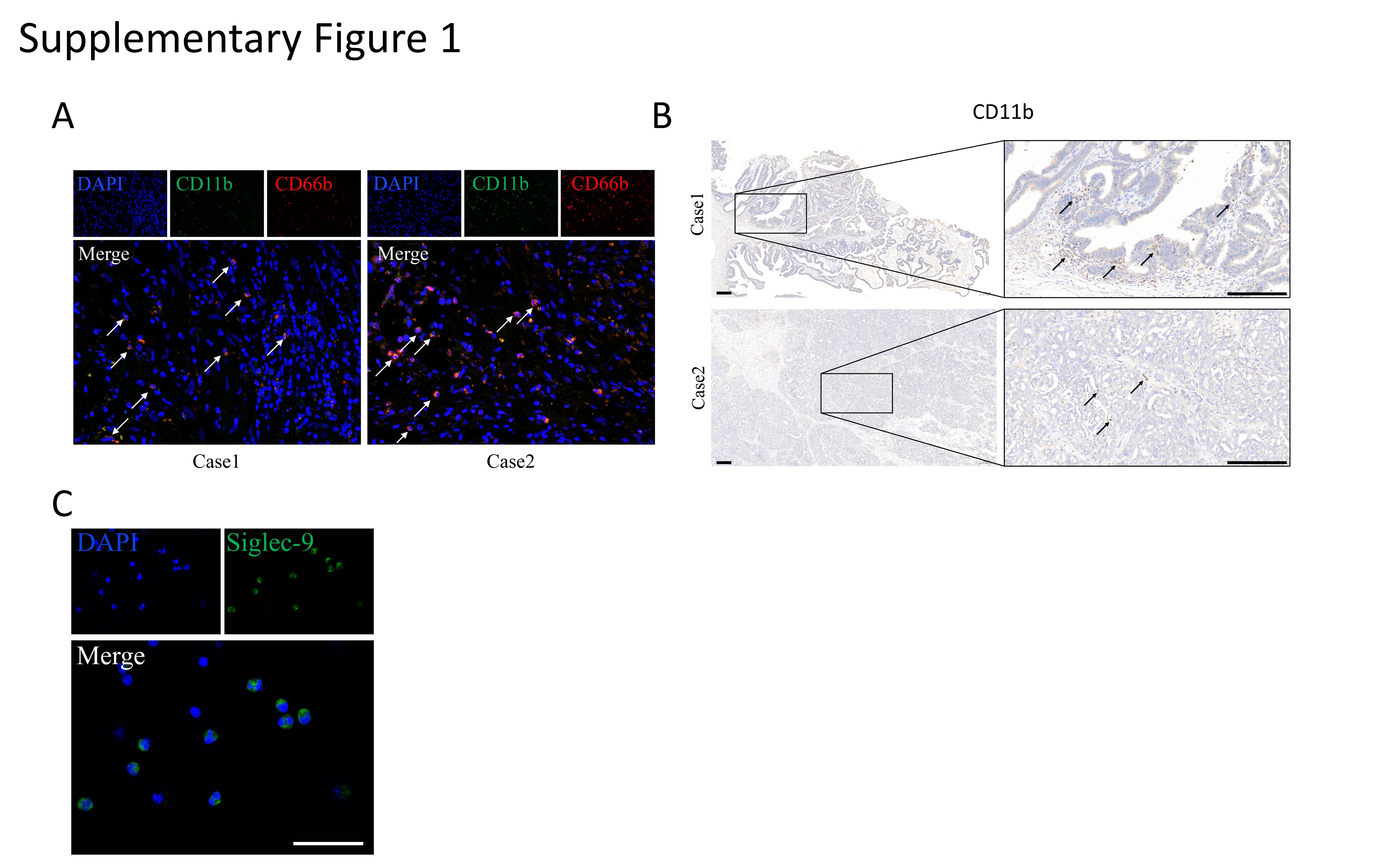


Supplementary Figure 1. The infiltration of neutrophils in ovarian cancer tissues and the expression of Siglec-9 on neutrophils. (A) Immunohistochemical staining of neutrophils markers CD11b and CD66b in ovarian cancer tissues. (B) Immunofluorescence of neutrophils markers CD11b in ovarian cancer tissues. Bar=200μm. (C) The expression of Siglec-9 on neutrophils. Bar=50μm.


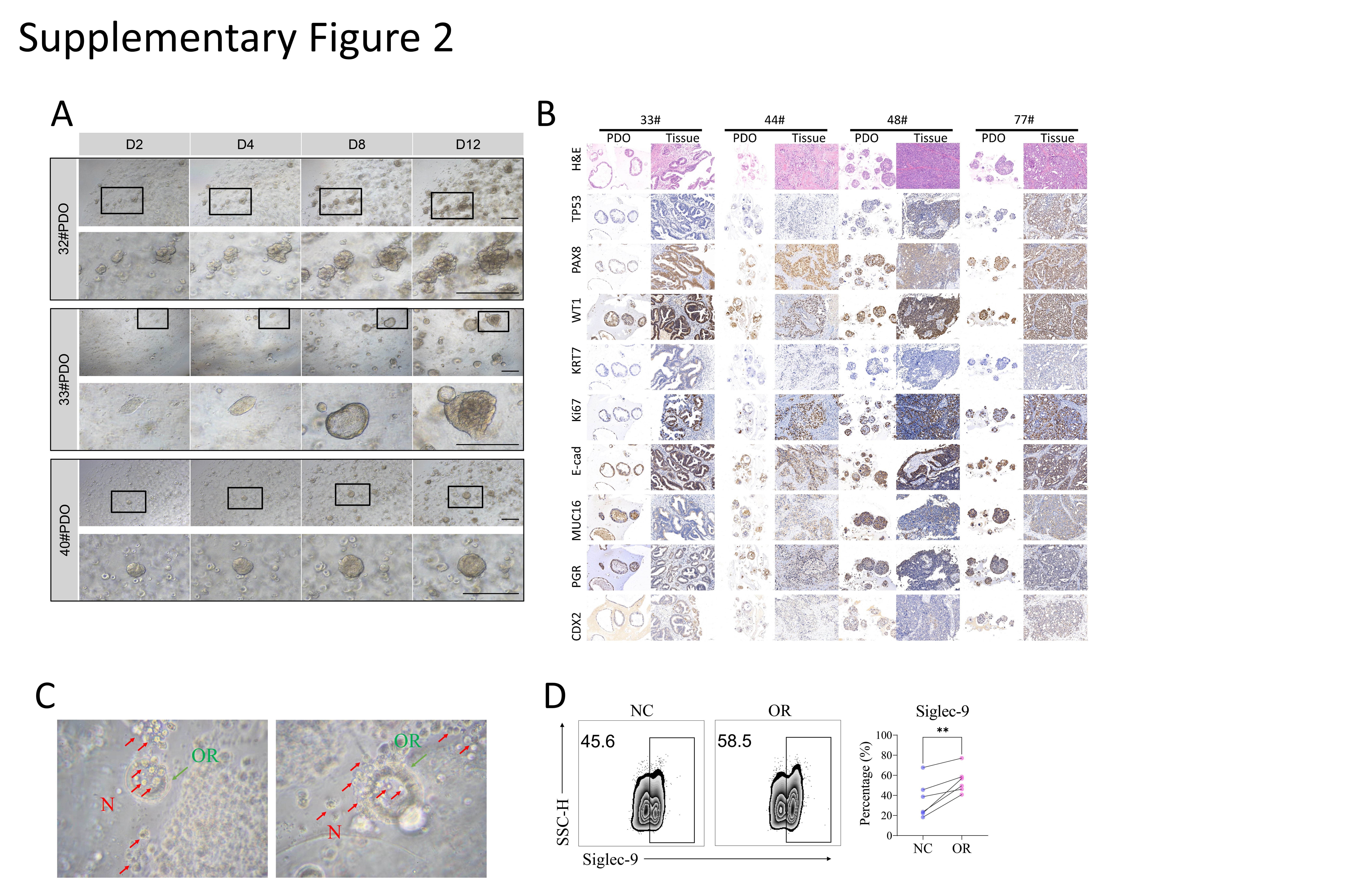


Supplementary Figure 2. The establishment of ovarian cancer organoid and stimulation to neutrophils. (A) Representative images of ovarian cancer organoids cultured to days 2, 4, 8, and 12. PDO: Patient-derived ovarian cancer organoids. Bar=1000μm. (B) H&E and IHC staining of representative ovarian cancer organoids. (C) Co-culturing of ovarian cancer organoids and neutrophils for 24h. Red arrows point to neutrophils and green arrows point to ovarian cancer organoids. Bar=50μm. (D) Siglec-9^+^ neutrophils in ovarian cancer organoid stimulation group (OR) and the control group (NC). The left panel showed the representative flow cytometry results, and the right panel showed the experimental results of 5 cases of neutrophils from different people. Paired T-test, **p<0.01.


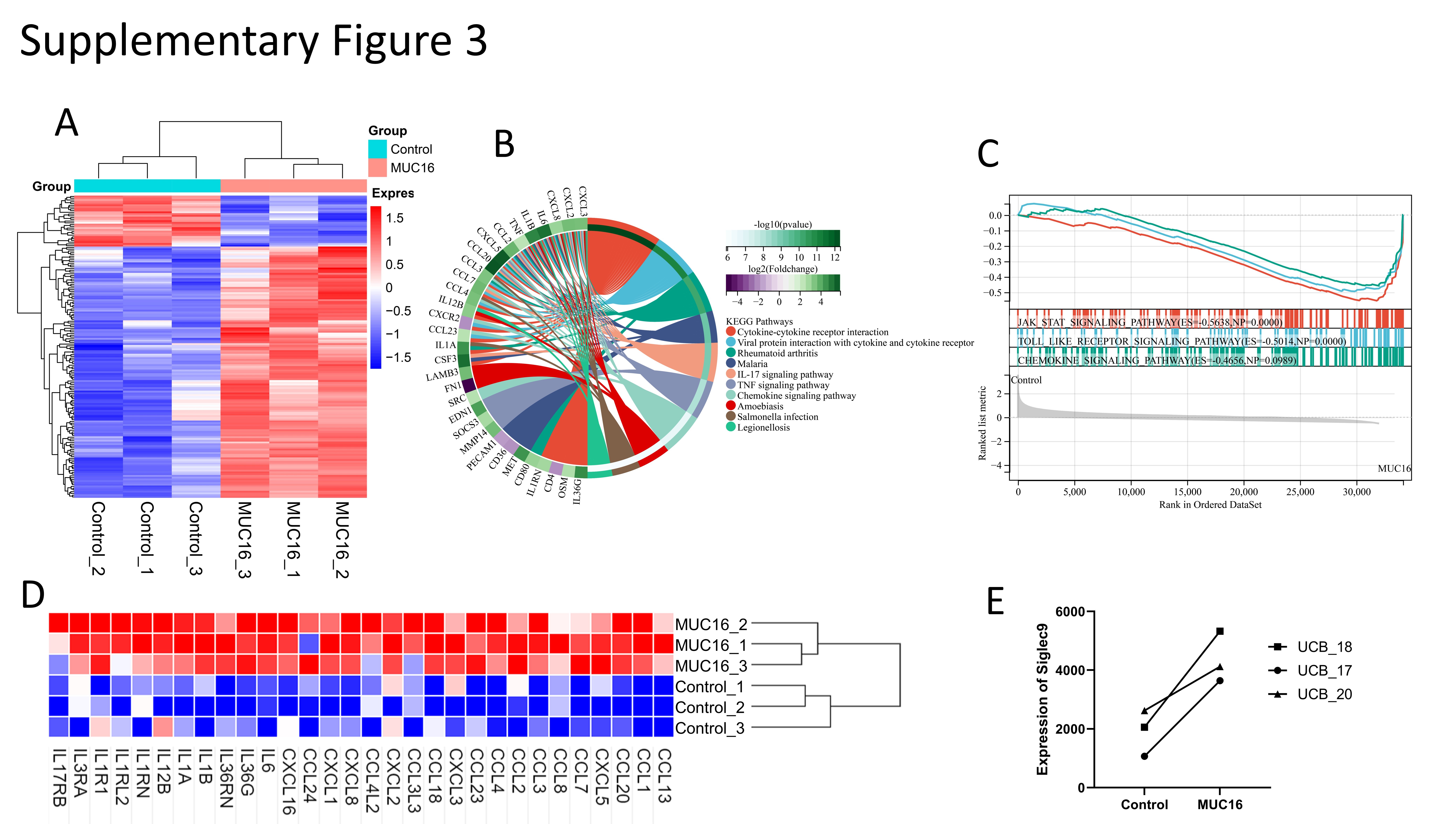


Supplementary Figure 3. The RNA-sequencing analysis of MUC16-treated neutrophils. (A) Heat map of differentially expressed genes (|log2FC|>2, p<0.05) in the MUC16-treated group compared to the control group. (B) Circos of KEGG enrichment analysis of differentially expressed genes in the MUC16 treatment group compared to the control group. (C) Results of GSEA analysis according to KEGG in the MUC16 treated group compared to the control group. (D) Heat map of chemokines and receptors and Interleukins. (D) Siglec-9 expression in MUC16-treated neutrophils and the control group.

**Supplementary Tables**

**Supplementary Table 2.** Reagents and antibodies in the research.

| **Application** | **Regent/Antibody** | **Company** | **LOT** |
| --- | --- | --- | --- |
| Organoid culture | Advanced DMEM/F12 | Thermo Fisher | 12634028 |
|  | Glutamax | Thermo Fisher | 35050061 |
|  | HEPES | Thermo Fisher | 15630080 |
|  | Nicotinamide | Sigma-Aldrich | N0636 |
|  | N-acetyl L-cysteine | Sigma-Aldrich | A9165 |
|  | Noggin | Novoprotein | CB89 |
|  | A8301 | Sigma-Aldrich | SML0788 |
|  | R-spondin-1 | Novoprotein | CX83 |
|  | FGF10 | Novoprotein | CR11 |
|  | EGF | Peprotech | 100-15 |
|  | Heregulinβ-1 | Peprotech | 100-03-50 |
|  | Foskolin | R&D | 1099 |
|  | Y27632 2HCl | Selleck | S1049 |
|  | β-Estradiol | Sigma-Aldrich | E2758 |
|  | B27 | Thermo Fisher | 17504044 |
|  | Penicillin-Streptomycin (PS) | Thermo Fisher | 15140122 |
|  | Matrigel | Corning | 354230 |
|  | TrypLE™ Express | Thermo Fisher | 12605028 |
|  | Cell recovery solution | Corning | 354253 |
| Flow Cytometry | CD11b Monoclonal Antibody (ICRF44), PE | eBioscience™ | 12-0118-42 |
|  | Mouse IgG1 kappa Isotype Control (P3.6.2.8.1), PE | eBioscience™ | 12-4714-82 |
|  | Alexa Fluor® 647 Mouse Anti-Human CD66b | BD | 561645 |
|  | Alexa Fluor® 647 Mouse IgM, κ isotype control | BD | 560806 |
|  | FITC Mouse Anti-Human CD329 (Siglec-9) | BD | 550906 |
|  | FITC Mouse IgG1, κ Isotype Control | BD | 555748 |
|  | BV421 Mouse IgG1, k Isotype Control | BD | 562438 |
|  | BV421 Mouse Anti-Human CD54 | BD | 564077 |
|  | APC Mouse Anti-Human CD184 | BD | 560936 |
| ELISA | 人CA125 ELISA kit | Abclonal | RK01022 |
| ROS detection | DCFH-DA | Sigma-Aldrich | D6883 |
| Immunohistochemistry/  Immunofluorescence | Anti-Ki67 | Thermo Fisher | PA1-21520 |
|  | Anti-KRT7 | Abclonal | A2574 |
|  | Anti-PAX8 | Abcam | ab53490 |
|  | Anti-CDX2 | Servicebio | GB121500 |
|  | Anti-WT1 | Servicebio | GB11382 |
|  | Anti-E-cad | Servicebio | GB13083 |
|  | Anti-TP53 | Abcam | ab1101 |
|  | Anti-Siglec-9 | Abcam | ab96545 |
|  | Anti-CD11b | Abcam | ab133357 |
|  | Anti-CD66b | Abcam | ab197678 |
| Cytotoxicity Assay | LDH Cytotoxicity Assay Kit | Beyotime | C0017 |
| Stimulation | Recombinant Human MUC16 (CA125) | Novoprotein | CW57 |

**Supplementary Table 3.** Primers used in the research.

| Gene | F/R | Sequence |
| --- | --- | --- |
| IL8 | F | GCTCTGTGTGAAGGTGCAGTTT |
|  | R | TTCTGTGTTGGCGCAGTGT |
| TNF-a | F | CCGAGTGACAAGCCTGTAGC |
|  | R | AGGAGGTTGACCTTGGTCTG |
| MMP9 | F | ACGTCTTCCAGTACCGAGAG |
|  | R | GGCACTGCAGGATGTCATAG |
| IL1B | F | TACGAATCTCCGACCACCA |
|  | R | GGACCAGACATCACCAAGC |
| VEGF | F | CCATTGTGGAGGCAGAGAAA |
|  | R | GATCAGGGAGAGAGAGATTGGA |
| CXCR2 | F | CAGCGACCCAGTCAGGATTTA |
|  | R | ACCAGCATCACGAGGGAGTTT |
| OSM | F | CACAGAGGACGCTGCTCAGT |
|  | R | GCTGGTGTCCTGCATGAGA |
| CXCL3 | F | CGCCCAAACCGAAGTCATAG |
|  | R | GCTCCCCTTGTTCAGTATCTTTT |
| MPO | F | CACCCTCATCCAACCCTTCATGTT |
|  | R | CATGTTCAGAGCAGGCAGGTCCAG |
| ARG1 | F | GTGGAAACTTGCATGGACAAC |
|  | R | AATCCTGGCACATCGGGAATC |
| β-actin | F | GTGGCCGAGGACTTTGATTG |
|  | R | AGTGGGGTGGCTTTTAGGATG |
| GAPDH | F | GTCGGAGTCAACGGATTTGG |
|  | R | CGGTGCCATGGAATTTGCC |
